# Diagnostic and therapeutic determinants in parathyroid surgery: A retrospective cohort study

**DOI:** 10.1016/j.amsu.2022.104751

**Published:** 2022-09-27

**Authors:** Achraf Amine Sbai, Adil Abdenbi Tsen, Fahd Elayoubi

**Affiliations:** a Department of Ear Nose and Throat, Mohammed VI University Hospital, Medical School, Mohammed the First University, Oujda, Morocco; b Laboratory of Epidemiology, Clinical Research and Public Health, Faculty of Medicine and Pharmacy of Oujda, Mohammed the First University, Morocco; c Department of Cervicofacial Surgery, Mohammed VI University Hospital, Medical School, Mohammed the First University, Oujda, Morocco

**Keywords:** Hyperparathyroidism, Vitamin D, Body mass index, Parathyroid adenomas

## Abstract

**Introduction:**

The systematic realization of biochemical and radiological examinations of the parathyroid has increased the incidence of primary hyperparathyroidism; which explains the increasing incidence of parathyroid surgery. Our study aims to predict the factors determining the diagnosis and management of parathyroid adenomas.

**Methods:**

We included 87 patients who presented with hyperparathyroidism and underwent parathyroid surgery. Ultrasound, computed tomography (CT), and more rarely sestamibi, were performed to localize the lesion preoperatively. Body mass index (BMI), blood and urine calcium and PTH concentrations before and after surgery, and preoperative vitamin D concentrations were evaluated.

**Results:**

In 90.8% of the cases, the location of the adenomas was retained thanks to cervical ultrasound, and in 86% of the cases, the ultrasound results were concordant with the intraoperative results, whereas the MIBI scanner was used in only 6 patients with a specificity and sensitivity of 100%, but these results cannot be taken into account because the sample is too small. No significant association was found between weight and preoperative vitamin D concentration, whereas we find a positive correlation between preoperative vitamin D concentration and adenoma weight (p = 0.001). Postoperative follow-up showed a positive relationship between the onset of hypocalcemia and vitamin D deficiency, and an inverse correlation between PTH concentration and postoperative hypocalcemia. All patients with an unknown vitamin profile (n:4) who developed postoperative hypocalcemia had a BMI greater than 25 kg/m2. The higher the PTH concentration in the preoperative period, the more profound the hypocalcemia.

**Conclusion:**

Further studies are needed to explore the role of vitamin D in the localization of parathyroid adenomas on the one hand, and to properly document the association between BMI and preoperative PTH concentration on the other.

## Highlights

• We report our experience in predicting the factors determining the diagnosis and management of parathyroid adenomas.
• We report for the first time the correlation between BMI and preoperative PTH.
• We also report for the first time the correlation between BMI and the occurrence of postoperative hypocalcemia.

## 1 Introduction

Primary hyperparathyroidism is an endocrine disorder that affects 0.3% of the general population and is more common in women than in men. It is responsible for an autonomous hypersecretion of parathyroid hormone (PTH), and subsequently for an overproduction of calcium. In 85% of cases, hyperparathyroidism is caused by a solitary parathyroid adenoma, in other cases it may be secondary to multiglandular disease, while parathyroid carcinoma is a much rarer cause of hyperparathyroidism [1,2].

The diagnosis of hyperpatahyroidism is easily made, it is often diagnosed in asymptomatic patients on routine biochemical tests. while the challenge is to identify the correct location of parathyroid adenomas. The localization of parathyroid adenomas is most often established by the use of several radiological examinations, including ultrasound (USG), computed tomography (CT), and 99mTc sestamibi (MIBI) scintigraphy, but sometimes the correct localization is only done intraoperatively [1,3].

Surgery is the first therapeutic option for most patients. It provides relief of various specific (renal and bone symptoms) and nonspecific (nonspecific musculoskeletal signs and neurocognitive and psychiatric disorders) symptoms of the disease. clinically and biologically advanced primary hyperparathyroidism is often associated with vitamin D deficiency. this deficiency may be incriminated by some authors in the occurrence of starved bone syndrome (postoperative hypocalcemia) despite normal postoperative parathyroid hormone concentrations, but this hypothesis remains, but other authors have not found a significant association between postoperative hypocalcemia and vitamin D deficiency. however, replacement of preoperative vitamin D deficiency is recommended [1].

In this paper, we analyzed the concordance of preoperative PTH and vitamin D concentration with other clinical, biological and radiological parameters and even intraoperative data, in order to identify the diagnostic and therapeutic determinants in hyperparathyroidism.

## 2 Material and methods

This is a prospective observational study carried out in the ENT department at the mohamed VI Hospital, OUJDA, morocco. Data were collected from patients who were treated surgically between 2015 and 2022 for primary hyperparathyroidism by the same surgical team.

We evaluated preoperative data, such as age, sex, BIM, symptoms and complications, blood and urine calcium concentrations, serum PTH, and vitamin D (when available), and collected postoperative biochemical results.

We evaluated preoperative data, such as age, sex, symptoms and complications, blood and urine calcium concentrations, serum PTH, and vitamin D (when available), and collected postoperative biochemical results (Table 1).

**Table 1:** Patient characteristics.

Number of patients		87
Sexe	Men	9
	Women	78
Mean age		62,51 (49–74) years
body mass index (kg/m2)	less than 18	3
	18.5–24.9	40
	25–29.9 9	36
	≥30	8
Symptoms et complications	Bone complication	19
	Renal complication	14
	Other complications	18
	No disease-specific symptoms	36
Mean preoperative PTH concentration (reference at local laboratory 1.8–7.4 pmol/L)		34 (8–243)
Mean preoperative calcium concentration (reference range at local laboratory 2.3–2.8 mmol/L)		2.9 (2.6–3.74)
Mean urine calcium concentration (reference range at local laboratory 100–250 mg/24 H)		255 (190–400)
Mean Vitamine D concentration (reference range at local laboratory 30–70 ng/mL)		22 (4–45)
Localization of adenomas on imaging	Ultrasound scan	79
	CT	61
	Sestamibi	6
	Peropératoire	2
Medical treatment	Venous rehydration	10
	Bisphosphonate	5
	calcium-mimetic	0
Surgery	Adenectomy	81
	Subtotal resection	6
weight of the adenomas (gram)		1,4 (0,002–10)
Mean postoperative PTH concentration (reference at local laboratory 1.8–7.4 pmol/L)		4.6 (1.9–12)
Average postoperative calcium concentration (reference range at local laboratory 2.3–2.8 mmol/L)		2.5 (1,9–3)
Surgical recovery		4

We evaluated the factors that may influence the visualization of the preoperative location of the adenoma obtained by ultrasound, and compared them to the actual intraoperative findings. In case of doubt, we will use CT of the neck and mediastinum and more rarely scintigraphy (problem of cost and availability).

Patients with unilateral adenoma with accurate location on imaging were operated on through a neck access. While for patients with unlocalized tumors or in case of doubt, they underwent bilateral neck exploration. We noted the locations found on preoperative imaging and intraoperative findings to study concordance.

Baseline PTH was taken 30 min after removal of the adenoma and compared with intraoperative PTH. Success of the operation is defined by a drop in PTH from baseline, when PTH does not drop we repeated the PTH measurement and explored the neck, we weighed the specimens and confirmed the nature of the adenoma by anatomopathological study.

In this paper, we studied the impact of several clinical biological and radiological parameters on the concentration of vitamin D and preoperative PTH, the analysis of all these data allowed us to compare our results with the literature to predict the diagnostic determinants (especially the location) and the management (the success rate) of surgical hyperparathyroidism.

The ethical review committee at faculty of medicine, Mohammed the First University of Oujda (CERBO), approved the study design and protocol. our study has been reported following the STROCS 2021 criteria [4].

## 3 Results

### 3.1 Preoperative exploration (clinical and biochemical)

A total of 87 patients were included in this study (median age 62.51 (range 49–74) years). Nine (11%) of the patients were male and (89%) were female. 50.6% (n:44) of patients had a BMI greater than 25 kg/m2. 51 of the patients were symptomatic (57.5%) of which 37% (n = 19) had bone manifestations, although 24 (42.5%) had no specific symptoms of hypercalcemia.

PTH concentrations were elevated with a mean of 34 (8–243) (local laboratory reference 1.8–7.4 pmol/L). Most of our patients had increased calcium excretion, we also find inappropriately increased concentrations of preoperative blood calcium with a mean of 2.9 (2.6–3.74) pmol/L (local lab reference 2.3–2.8 mmol/L).

Preoperative vitamin D concentrations were measured in 61 (70%) of the patients (mean preoperative concentration 22 (4–45) nmol/L; laboratory reference range 75–200 nmol/L), however in 30% of the patients the vitamin D profile was unknown.

In vitamin D-deficient patients PTH concentrations were higher, suggesting an inverse linear relationship between preoperative vitamin D and PTH concentrations (p = 0.012).

A positive correlation was found between BMI and PTH concentration, patients with increased BMI had higher preoperative PTH levels (p: 0.033). While an inverse correlation was found between BMI and vitamin D concentration, obese and overweight patients had significantly more vitamin D deficiency (p: 0.04). we also find a correlation between the concentration of vitamin D, PTH and bone manifestations, we note the presence of bone symptoms especially in patients with vitamin D deficiency (p: 0.04), and present high concentrations of PTH (p: 0.03). While no correlation was found between vitamin D, PTH concentration, and blood and urine calcium concentration.

To control calcium concentration before surgery, 10 patients (11%) received intravenous rehydration and 5 patients (5.5%) were treated with bisphosphonates (Table 2, Table 3).

**Table 2:** Patient characteristics for patients with and without vitamin D deficiency.

	All patients	with vitamin D deficiency	without vitamin D deficiency	vitamin D dosage not done	P value
Number of patients	87	33	28	26	–
Age (years) [median]	62,51	61,1	58,4	62,9	0,432
Sex [n %]					
Women	78	29	26	23	
Men	9	4	2	3	0,765
Body mass index (kg/m2)					0,04
less than 18	3	0	3	0	
18.5–24.9	40	9	15	10	
25–29.9 9	36	16	7	11	
30-35	8	4	1	3	
>35					
Symptoms et complications:					
Bone complication	19	10	3	6	0,04
Renal complication	14	5	4	5	0,294
Other complications	18	9	6	3	0,295
No disease-specific symptoms	36	10	12	14	0,461
Mean preoperative PTH concentration (reference at local laboratory 1.8–7.4 pmol/L)	34	41	28	39	0,012
Mean preoperative calcium concentration (reference range at local laboratory 2.3–2.8 mmol/L)	2.9	3,4	3,5	2,8	0,146
Localization of adenomas					
Ultrasound scan	79	28	13	20	0,410
CT	61	23	19	19	0,345
Sestamibi	6	5	1	0	0,03
Peroperative	2	1	1	0	0,187
Mean postoperative PTH concentration (reference at local laboratory 1.8–7.4 pmol/L)	4,6	5,6	6	4,1	0,376
Average postoperative calcium concentration reference range at local laboratory 2.3–2.8 mmol/L)	2,5	2,5	2,4	2,6	0,429
Average weight of the adenomas (gram)	1,4	0,9	1,3	1,5	0,345
postoperative hypocalcaemia (nombre)	16	10	2	4	0,001

**Table 3:** Patient characteristics by preoperative PTH concentration.

	All patients	Mean preoperative PTH concentration (<10 pmol/L)	Mean preoperative PTH concentration (>10 pmol/L)	P value
Number of patients	87	51	36	–
Age (years) [median]	62,51	63,3	60,09	0,167
Sex [n %]				
Women	78	45	33	0,311
Men	9	6	3	
Body mass index (kg/m2)				
less than 18	3	3	0	0,033
18.5–24.9	40	36	4	
25–29.9 9	36	10	26	
30-35		2	6	
>35	8			
Symptoms et complications:				
Bone complication	19	4	15	0,03
Renal complication	14	7	7	0,280
Other complications	18	10	8	0,167
No disease-specific symptoms	36	24	12	0,02
Mean Vitamin D cencetrartion	22	21	6,5	0,012
Mean preoperative calcium concentration (reference range at local laboratory 2.3–2.8 mmol/L)	2.9	3	3,5	0,147
Localization of adenomas				
Ultrasound scan	79	29	30	0,260
CT	61	19	23	0,457
Sestamibi	6	4	2	0,09
Intraoperative finding	2	2	0	0,07
Mean postoperative PTH concentration (reference at local laboratory 1.8–7.4 pmol/L)	4,6	5,8	2,6	0,01
Average weight of the adenomas (gram)	1,4	0,3	3,3	0,001
Average postoperative calcium concentration reference range at local laboratory 2.3–2.8 mmol/L)	2,5	2,3	2,71	0,012
postoperative hypocalcaemia (nombre)	16	4	12	0,001
less than 18				
18.5–24.9				
25–29.9 9				
30–35				
>35				

### 3.2 Diagnosis of location

There was no significant association between preoperative PTH concentrations, vitamin D, and radiological detectability of adenomas. In 90,8% of the cases the location of the adenomas was retained thanks to the cervical ultrasound, and in 86% of the cases the results of the ultrasound were concordant with the intraoperative findings, while the MIBI scan was used in only 6 patients with a specificity and sensitivity of 100%, but these results cannot be considered since the sample is too small.

While in 2.3% of patients (n:2) surgical exploration was used to visualize the adenoma.

All patients underwent preoperative sestamibi and ultrasound examinations. A total of 29 (25%) required a CT scan for further evaluation. Preoperative sestamibi yielded true-positive results in 95 cases (81%) and false-positive results in 12 cases (10%). The sestamibi yielded a false-negative result in eight cases (7%), and in two cases (2%), the adenoma was not identified on the MIBI or during surgery (true negative). The sensitivity of the sestamibi scan was calculated as 92% with a positive predictive value of 88%. The specificity was 14% with a negative predictive value of 20%.

In 84 cases (72%), the ultrasound results were truly positive and matched the intraoperative results. The mean weight of the adenomas was 1.4 g (0.002–10 g). There was no significant association between weight and preoperative vitamin D concentration, whereas we find a positive correlation between preoperative vitamin D concentration and adenoma weight (p = 0.001). (Table 2, Table 3).

### 3.3 Postoperative exploration

All patients were seen in ENT consultation 6 weeks after the operation, in 86 patients (98.9%) the calcium concentration had returned to normal, hypercalcemia persisted in only one patient even though histological examination of frozen sections confirmed that the specimens were parathyroid tissue, in this patient a surgical revision is planned. There was a concordance between preoperative and postoperative PTH concentration, the higher the preoperative PTH, the lower the postoperative PTH after removal of the adenoma (p < 0.01). In 2 patients (2,29%), the adenoma could not be found preoperatively while they were identified intraoperatively, after their removal the PTH decreased sufficiently and the calcium concentrations returned to normal.

Sixteen patients (18.4%) had transient postoperative hypocalcemia (range 1.9–2.1), there was a positive relationship between the occurrence of hypocalcemia and vitamin D deficiency, and an inverse correlation between PTH concentration and postoperative hypocalcemia. All patients with unknown vitamin profile (n:4) who presented hypocalcemia postoperatively had a BMI greater than 25 kg/m2. The higher the PTH concentration in the preoperative period, the deeper the hypocalcemia (Table 2, Table 3).

## 4 Discussion

Concerning the concordance between preoperative vitamin D concentrations and PTH concentration (p: 0,012), our results are similar to those of the literature [1,5]. While in this paper we will analyze all the data whether clinical, biological, radiological or operative with these two parameters (vitamin D and preoperative PTH).

No significant association was found between the onset of clinical symptoms and the concentration of vitamin D and PTH preoperatively, except for bone symptoms and complications whose incidence increased with increasing PTH (p:0,04) and decreasing vitamin D (p:0,03). It is known that hyperparathyroidism has adverse effects on bone [6]. while vitamin D deficiency is incriminated in osteoporosis [7]. Vitamin D deficiency in hyperparathyroidism may give a synergistic effect on bone damage.

In our study the localization of the parathyroid adenoma was essentially done with the cervical ultrasound, and the sestamibi was used only in 6 patients with a high sensitivity and specificity, our results are in agreement with the literature [3,8].

A significant association is found between the location of the adenoma by seta mibi and vitamin D deficiency, this result has more value in studies with a greater number of scans performed. The concentration of preoperative PTH reflects the weight of the parathyroid adenoma, this concordance has already been reported by other studies. while there is no concordance between the weight of the adenoma and preoperative vitamin D [1,9].

Comparing the postoperative blood calcium concentration with preoperative PTH and vitamin D, no significance was found with vitamin D, however in the literature and even in the current study a significant association was found with preoperative PTH (p: 0,012) [1].

The majority of the 16 patients who presented with postoperative hypocalcemia had a very high preoperative PTH (p) and/or a vitamin D deficiency, which can be explained by the hungry bone syndrome [10]. Our study is the first to include BMI as a determinant of hyperparathyroidism. There is an inverse association between BMI and vitamin D concentration, which has been reported in several studies [11]. While in our study we also find a positive correlation between BMI and preoperative PTH concentration, this can be explained by the vitamin D deficiency in patients with a high BMI, which will lead to an increase in PTH. There is also a correlation between BMI and postoperative hypocalcemia, the explanation of which is always related to the deficiency of the vitamin in patients with high BMI. The limitations of our study are that 27.5% of the patients did not have vitamin D, other studies in the future may better explain the relationship between the concentration of PTH and BMI.

The correlation between BMI and preoperative PTH and BMI and the occurrence of postoperative hypocalcemia, can be explained by the level of vitamin D, which is correlated to BMI. On the one hand, patients with a high BMI have a low level of vitamin D, which has been proven before, and on the other hand, several authors have reported a correlation between the preoperative PTH level and vitamin D (an inverse correlation), and a correlation between the occurrence of postoperative hypocalcemia and the level of vitamin D (a positive correlation): the lower the vitamin D, the more frequent the postoperative hypocalcemia) [10,11], in front of these data it was concluded that the correlation between BMI and preoperative PTH and BMI and the occurrence of postoperative hypocalcemia is the result of the concentration of vitamin D that varies in patients according to their BMI. To our knowledge, we are the first to study BMI as a determinant factor in the diagnosis and therapeutic management of parathyroid adenoma, but more studies are needed to elucidate the role of BMI in parathyroid adenoma.

## 5 Conclusion

In summary, this study reported several significant associations between several clinical, biological, radiological, and operative parameters these results were already reported by other authors. However, BMI was found to have a significant association with preoperative PTH concentration on the one hand and with the occurrence of postoperative hypocalcemia on the other hand, we suggested that this association is related to vitamin D deficiency in patients with high BMI, further studies are desirable to confirm our results.

## Ethical approval

The ethical review committee at faculty of medicine, Mohammed the First University of Oujda (CERBO), approved the study design and protocol. Data were anonymously registered in our database. Access to data was approved by the head of the department.

## Please state any sources of funding for your research

This research was not funded.

## Author contributions

Dr. Achraf Amine SBAI wrote the manuscript.

Pr. Adil Abdenbi Tsen helped in writing and literature review.

Pr. Fahd ELAYOUBI helped in writing, supervised the redaction, revised and approved the final draft for publication.

All authors approved the final version of the manuscript.

## Registration of research studies

UIN: 7745.

## Guarantor

Dr Achraf SBAI.

## Consent

Written informed consent was obtained from patients for the publication of their cases. A copy of the written consents is available upon request for review by the editor of this journal.

## Provenance and peer review

Not commissioned, externally peer reviewed.

## Please state any conflicts of interest

The authors declare no conflicts of interest.
